# Establishment of a Native Cavity‐Nesting Bee (*Exoneura robusta*) After Translocation Into an Urban Environment

**DOI:** 10.1002/ece3.73567

**Published:** 2026-04-30

**Authors:** Mulan Wang, Julian Brown

**Affiliations:** ^1^ School of Agriculture, Food and Ecosystem Sciences The University of Melbourne Melbourne Australia

## Abstract

Urban environments are increasingly considered as potential sites for pollinator conservation, yet quantitative evaluations of insect translocations into cities remain limited. We report the outcome of a small‐scale translocation of the native cavity‐nesting bee *Exoneura robusta* from forest habitat into an urban campus in Melbourne, Australia. Adult bees from natural nests were relocated into artificial balsa‐wood nests and installed across shaded and open microhabitats within an urban greenspace. Nest‐level success was defined as the presence of brood in the first summer following translocation and the presence of adults the following winter, indicating completion of a full annual cycle. We hypothesized that the native cavity‐nesting bee *Exoneura robusta* can successfully establish in urban environments following translocation, and that nest success is associated with initial adult group size, canopy cover, or local floral richness. Of the eleven artificial nests established, six met this criterion for success. We used binomial generalized linear models to test whether initial adult group size, canopy cover, or local floral richness were associated with nest success. None of the measured variables showed strong support relative to a null model, suggesting that simple nest‐level predictors did not explain variation in establishment outcomes within this small experimental dataset. Successful nests occurred across a range of urban microhabitats, including open sites adjacent to built infrastructure and locations beneath dense canopy. Our findings demonstrate that *E. robusta* can persist for at least one annual cycle following translocation into an urban environment. Although limited in scale, this study provides initial evidence that native cavity‐nesting bees can establish within compact urban greenspaces and highlights the need for larger experiments incorporating finer‐scale biotic and microclimatic measurements.

## Graphical abstract

We translocated the native cavity‐nesting bee Exoneura robusta into an urban greenspace using artificial nests to test short‐term establishment success. Five of eleven nests persisted through a full annual cycle, demonstrating that urban environments can support early colony establishment. Simple nest‐level predictors showed no strong support, highlighting the complexity of establishment processes in urban settings.

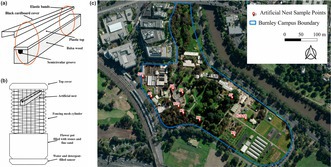

## 1 Introduction

Pollinators play a central role in terrestrial ecosystems and food production (Jones and Rader 2022; Duarte et al. 2024), yet numerous pollinator taxa have declined in recent decades due to habitat loss, climate change, pesticides, and pathogens (Bartomeus et al. 2013; Goulson et al. 2015; Sánchez‐Bayo and Wyckhuys 2019; Ramos‐Jiliberto et al. 2020). Urbanization adds further pressure by fragmenting habitats and altering the timing and continuity of floral resources (Wenzel et al. 2020). Despite these challenges, urban environments are increasingly recognized as potential refuges for pollinator conservation due to their management potential and floral diversity (Hall et al. 2017; Baldock et al. 2019). While habitat restoration is common, active interventions such as translocation are widely used for vertebrates yet remain quantitatively underevaluated for insects in urban settings (Watson and Watson 2015; van Heezik and Seddon 2018; J. Brown et al. 2024).

For cavity‐nesting bees, substrate availability (e.g., dead wood and hollow stems) is often a limiting factor in managed urban landscapes compared to natural forests. Translocating social or semi‐social insects presents specific challenges, including the need to move entire social units and a critical dependence on immediate resource availability to prevent failure (Sheffield et al. 2008; M. J. F. Brown et al. 2017; Folly et al. 2025). Artificial nesting structures such as balsa‐wood nests overcome these substrate limitations while enabling monitoring of establishment success (Schwarz and Overholt 1993). *Exoneura robusta* is a primitively social, stem‐nesting bee native to southeastern Australia that forms small cooperative groups and forages on a wide range of native and exotic flowers (Repaci et al. 2006; J. Brown et al. 2020; Coates et al. 2022). Historical records indicate long‐term persistence in Melbourne's urbanizing landscapes (Victorian Department of Environment, Land, Water and Planning 2025), yet its reliance on discrete nesting substrates and pronounced seasonal cycle make establishment sensitive to local microclimate, floral resources and founding group size (Cronin and Schwarz 1999a, 1999b; Landsman et al. 2019).

Here we report the outcome of a small‐scale translocation of *E. robusta* from native forest into an urban location. Our primary goal was to quantitatively evaluate nest‐level success, defined as colony persistence through a full annual cycle (brood production to overwintering adults). We hypothesized that the native cavity‐nesting bee *Exoneura robusta* can successfully establish in urban environments following translocation, and that nest success is associated with initial adult group size, canopy cover, and local floral richness. By testing these predictors, this study provides an initial assessment of the factors governing survival of native cavity‐nesting bees within the urban matrix.

## 2 Methods

### 2.1 Study Site and Translocation

**FIGURE 1:**
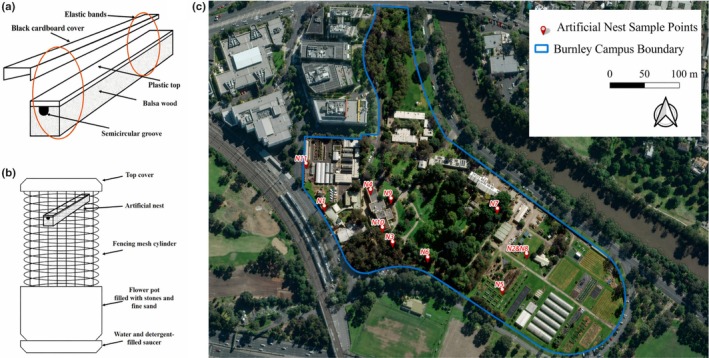
(a) Construction of artificial balsa nests and the plastic chambers, which can be attached to the nests. (b) Structure of the artificial bee tower used to house each nest. (c) Locations of the ten tower sites across the Burnley Campus (N2 and N8 were housed together in one tower).

This study was conducted at the University of Melbourne Burnley Campus, an urban greenspace with mixed garden beds, lawn areas and trees. In August 2024, we collected more than 30 stems containing candidate allodapine nests from wet eucalypt forest in the Dandenong Ranges. Candidate stems were identified by the presence of a~5 mm circular entrance hole and collected by clipping with pruning shears. After dissection in the laboratory, individuals identified as *Exoneura robusta* were allocated to 11 standardized artificial balsa‐wood nests (1.8 × 1.8 × 30 cm) with a 5 mm diameter semicircular groove (Figure 1a). To allow for non‐destructive observation, the groove was sealed with a transparent plastic top and a removable black cardboard cover, both secured with elastic bands (Figure 1a). Each translocation involved transferring the entire intact social unit found in the natural stem into the artificial nest, regardless of the specific sex ratio, whereas two individuals from separate low‐density natural nests were combined as was necessary.

The artificial nests were placed inside vertical wooden “bee towers” (Figure 1b) and installed at ten sites across the campus (Figure 1c) following the design developed by Schwarz and Overholt (1993). To mitigate predatory and competitive pressures, each tower was placed in a saucer of water with detergent to break surface tension (Schwarz et al. 1997). The placement was designed to cover two broad microhabitat categories:

Shaded tower sites: locations beneath dense vegetation with persistent overhead canopy (*n* = 5).

Open tower sites: locations fully exposed to sunlight with little or no canopy cover (*n* = 5).

These categories captured the major contrast in light and canopy structure present on the campus and provided a basis for evaluating whether microhabitat context influenced nest performance.

Following release, nest status was monitored at seven regular intervals, including the initial baseline (T0) recorded during translocation and six subsequent surveys. While we initially intended to rely on monthly non‐destructive surface inspections, *Exoneura robusta* began extensively excavating and remodeling the internal balsa‐wood structure as early as September 2024. By October, 50% of the nests exhibited significant excavation; as females prioritized brooding within these internal tunnels, surface‐level counts through the plastic cover became unreliable. To ensure accurate data collection while minimizing disturbance during the November breeding peak, the first comprehensive monitoring involving full nest disassembly was conducted in December 2024. In April 2025, a low‐intensity surface‐level inspection was performed to avoid excessive stress on the colonies heading into winter. A final comprehensive dissection was conducted in August 2025 to record survival and reproduction at the completion of a full one‐year cycle. Following each disassembly, all life stages were transferred into new balsa‐wood nests of identical specifications.

Three nest‐level variables were used as potential predictors. First, initial adult group size was recorded at the time of translocation, including counts of total, male and female adults. Second, canopy cover was estimated from hemispherical smartphone photographs taken directly above each tower. Images were processed using automated thresholding to calculate the proportion of sky blocked by vegetation. Third, floral richness was measured as the number of flowering entomophilous plant species within a 10‐m radius of each tower at the time of nest placement, which is critical for the progressive rearing requirements of this species (Schwarz et al. 1997).

### 2.2 Statistical Analysis

Nest‐level success was defined as a binary response. A nest was classified as successful if brood was present in December 2024 and at least one adult was present in August 2025. Nests that did not meet both conditions were classified as unsuccessful. Brood size was quantified during the December dissection as the total number of larvae, pupae, and callow adults. To account for the potential stress of nest monitoring, we treated the two comprehensive dissections as binary events in our success assessment, though all bees were immediately transferred back to identical nests to minimize impact.

To test whether any measured variable influenced success, we fitted a set of binomial generalized linear models (GLMs). Each model contained only one predictor variable. Predictor sets included initial adult group size, initial male adults, initial female adults, canopy cover, and floral richness. An intercept‐only GLM was used as a null model.

Models were compared using AICc to evaluate whether any predictor improved model fit relative to the null model. Given the small number of nests (*N* = 11), this single‐predictor structure avoided overparameterisation and provided a consistent basis for evaluating effect strength. All analyses were conducted in R Statistical Software (v4.4.0; R Core Team 2024) using the package MuMIn (Kamil Bartoń 2025).

## 3 Result

Eleven artificial nests were monitored between September 2024 and August 2025. Six nests (54.5%) successfully completed a full life cycle, containing brood in December and at least one adult in August. The remaining five nests did not meet both conditions and were classified as unsuccessful, with four failing during the initial three‐month establishment phase (prior to December 2024). In December, successful nests contained 2–19 brood individuals (larvae, pupae or callow adults), while unsuccessful nests contained none. Permanent food stores (provisions) were not recorded, as *E. robusta* is a progressive provisioner that does not stockpile mass provisions (Schwarz et al. 1997).

The binomial GLMs indicated only weak effects of the measured variables on nest success. The model containing initial adult group size received the most support (ΔAICc = 0.00, model weight = 0.354), followed closely by the intercept‐only null model (ΔAICc = 1.48). Models containing initial male/female counts, canopy cover, and floral richness all had ΔAICc values ranging from 1.05 to 3.88. The coefficient for initial adult group size was positive (*β* = 0.40) but not statistically significant (*p* > 0.05). Given the small sample size (*N* = 11), none of the measured nest‐level predictors had a dominant influence on the probability of completing a full annual cycle.

Success occurred under a range of local environmental conditions. Specifically, translocation was successful in highly modified environments, including two rooftop gardens (N4 and N10) and the edge of a busy car park (N11; Figure 1c). These observations suggest that factors unique to the urban landscape, such as extensive impervious surfaces, do not strictly prevent the establishment of *E. robusta* . Furthermore, the two comprehensive dissections did not trigger colony abandonment; colonies identified as active in December and April persisted through to the final August census.

## 4 Discussion

This study provides an initial evaluation of the short‐term establishment of a native allodapine bee within an urban greenspace. Six of the ten nest towers completed a full annual cycle, which indicates that translocation of *Exoneura robusta* into a highly modified environment is feasible. Successful nests occurred in locations typical of highly urbanized areas. This indicates that highly urbanized locations are capable of supporting the first generation of a transplanted population, consistent with evidence that many native bees can persist in small, heterogeneous habitat patches embedded within cities (Hall et al. 2017).

The binomial GLMs did not identify strong effects of initial adult group size, canopy cover or floral richness on nest success. This may reflect the complexity of bee nesting biology and the limited capacity of simple environmental metrics to capture this variation in a small experimental dataset. Urban bees experience fine‐scale variation in temperature, humidity and wind exposure that can alter brood development and adult survival, and these conditions are only partly related to canopy cover or coarse measures of floral richness (Davidowitz 2009; Abou‐Shaara et al. 2017; Hennessy et al. 2020). Cavity‐nesting bees are also influenced by conditions inside their nests. Species in the genus *Exoneura* excavate and modify their nesting substrate, which alters cavity geometry, internal insulation and moisture dynamics and therefore generates substantial variation in microclimate among nests that appear similar from the outside (Michener 1965; Antoine and Forrest 2021). These sources of biological and physical noise can mask relationships between measured environmental variables and nest‐level outcomes, especially when the number of experimental units is small.

During the analysis, we explored several continuous and behavioral metrics, including activity indices and excavation‐based measures, but these did not provide consistent or interpretable results. Measures based on short‐term activity and excavation intensity were highly variable across weeks and were strongly influenced by nest modification during the season. These patterns are consistent with studies showing that small social groups of allodapine bees can display marked plasticity in task allocation, provisioning, and brood investment (Cronin and Schwarz 1999a, 1999b; Cronin 2001).

Under such conditions, models that rely on detailed behavioral metrics are unlikely to perform well because the underlying processes are dynamic and only partially observed. In contrast, a simple binary endpoint that records whether a nest produced brood and retained adults into the following winter provides a robust summary of establishment over the full annual cycle. Binary indicators of establishment are commonly used in terrestrial insect reintroduction and translocation studies where sample sizes are modest and the primary question is whether released groups can persist through the early stages of colonization (Bellis et al. 2019).

In this context, the absence of strong effects should be interpreted as a cautious statement that we did not detect large, simple associations between our nest‐level predictors and success rather than as evidence that such associations do not exist.

Several unmeasured factors are likely to have contributed to variation in nest success. Predation by ants/spiders and occupation of cavities by other insects were observed in some nests, and these interactions can reduce brood survival independently of canopy conditions or flower richness. To mitigate ant predation, all towers were equipped with water and detergent saucers as described in Section 2.1. While opportunistic observations of other predators were noted, we did not include predation as a formal predictor in our models to avoid overparameterization given our small sample size (*N* = 11). Such biotic pressures have undermined otherwise well‐designed pollinator reintroductions in other systems, for example in the shorthaired bumblebee project where parasitoids and associated pathogens strongly affected establishment (M. J. F. Brown et al. 2017).

The composition of founding groups may also have played a role. While most artificial nests were formed from individuals originating from a single natural nest, in one instance, we combined individuals from two low‐density natural nests to ensure a viable founding group size. Social structure and relatedness influence division of labor, brood care and tolerance within allodapine colonies. However, given the high degree of social plasticity exhibited by allodapine bees, total adult group size is often a more critical driver of early nest success than specific relatedness, as the lack of cell partitions necessitates a constant adult presence to defend the communal gallery (Schwarz et al. 2007; Landsman et al. 2019). In our study, we prioritized group size over source type in our models to maintain statistical robustness within our limited dataset (*N* = 11).

The absence of a strong signal for initial group size is noteworthy, because theoretical work on reintroductions highlights the importance of founding population size and the potential for Allee effects at low densities (Courchamp et al. 1999; Stephens et al. 1999; Jamieson 2011). In *E. robusta* , females can found and maintain nests with small groups, and cooperative brood care is facultative rather than obligate (Schwarz et al. 2007). Our results are consistent with this view. Within the narrow range of group sizes used here, any benefits of larger founding groups may have been outweighed by individual variation in behavior, by predation or by nest usurpation. A stronger test of group size effects would require a wider and more controlled gradient in founding numbers and a larger number of nests.

Although our dataset is limited, the overall outcome remains informative for urban pollinator conservation. The presence of surviving adults in August in almost half of the nests demonstrates that *E. robusta* can persist for an entire annual cycle within a compact urban campus that contains both vegetated patches and hard surfaces. This suggests that the species has the capacity to tolerate the microclimatic and structural conditions associated with built environments, provided that some suitable nesting substrates and floral resources are available. From an applied perspective, our findings support the idea that small, well‐vegetated sites, including green roofs and edges of car parks, can contribute to the conservation of native cavity‐nesting bees, in line with broader arguments for reconciliation ecology in cities (Kidwell 2016; Baldock et al. 2019; Ward et al. 2022).

While our results provide a conservative baseline, several unmeasured anthropogenic and biological stressors likely govern translocation outcomes in urban matrices. Future research should prioritize testing these as explicit hypotheses: (1) Floral resource phenology: Beyond richness, “hungry gaps” or phenological mismatches in urban plantings may create critical resource bottlenecks; (2) Interspecific competition: High densities of managed honey bees ( *Apis mellifera* ) may lead to functional resource limitation through exploitative competition; (3) Biotic pressures: Specialist natural enemies, such as *Syntretus* parasitoid wasps, or secondary cavity occupiers like *Megachile*, may impose mortality independent of habitat quality; and (4) Anthropogenic anomalies: The impacts of urban pesticide exposure from residential gardening and artificial light at night (ALAN) on the circadian rhythms and foraging efficiency of native bees remain significant knowledge gaps. Framing these factors as testable hypotheses will move urban bee conservation from descriptive observations toward a predictive management framework.

Future work should combine experimental releases with larger sample sizes, more detailed microclimate monitoring and explicit tracking of predators and competitors within nests. These additions would improve estimates of the drivers of establishment and would help to identify which urban design features and planting schemes most effectively support translocated populations of *E. robusta* and other native bees.

## Author Contributions

**Mulan Wang:** data curation (lead), formal analysis (equal), investigation (equal), methodology (supporting), visualization (lead), writing – original draft (lead). **Julian Brown:** conceptualization (lead), methodology (lead), resources (lead), supervision (lead), writing – review and editing (lead).

## Funding

The authors have nothing to report.

## Conflicts of Interest

The authors declare no conflicts of interest.

## Data Availability

All the required data are available from Dryad at DOI: 10.5061/dryad.79cnp5jbf.
